# Efficacy of a reduced dose of DARUNAVIR/RTV in a cohort of antiretroviral-naïve and experienced HIV-infected patients: a medium-term follow-up

**DOI:** 10.7448/IAS.17.4.19822

**Published:** 2014-11-02

**Authors:** Massimiliano Lanzafame, Emanuela Lattuada, Fabio Rigo, Andrew Hill, Sandro Vento

**Affiliations:** 1 Infectious Diseases Unit, Department of Internal Medicine, “G.B. Rossi” Hospital, Verona, Italy; 2 Unit of Pharmacology, Department of Pharmacology and Therapeutics, University of Liverpool, Liverpool, UK; 3 Internal Medicine Unit, Department of Internal Medicine, Faculty of Medicine, University of Botswana, Gaborone, Botswana

## Abstract

**Background:**

The currently approved dose of darunavir/ritonavir is 800/100 mg once daily for PI-naïve patients, and 600/100 mg twice daily for PI-pretreated patients. However, in DRV-sensitive patients at baseline in the POWER 1/2 trials, similar rates of HIV RNA suppression (1 log reduction) were achieved with doses ranging from 400/100 mg once daily to 600/100 mg twice daily. In previously virologically suppressed patients, a reduced dose of DRV (600/100 QD) is non-inferior to the standard dose (800 mg QD)[1] and DRV concentrations in plasma and CSF are similar in patients receiving the above different doses [1, 2].

**Methods:**

Twelve treatment-naïve patients were started on darunavir/ritonavir 600/100mg once daily, with TDF/FTC (8) or ABC/3TC (4). Seven patients were switched to darunavir/ritonavir 600/100 mg once daily, with TDF/FTC (2), ABC/3TC (2), NVP (1), AZT/3TC (1). One was on monotherapy with DRV. Seven treatment-experienced patients were switched to darunavir/ritonavir 600/100 mg once daily, with TDF/FTC (5), ABC/3TC (1), RAL (1).

**Results:**

Of the 12 naïve patients (mean baseline HIV RNA 134,024 log10 copies/mL, range 4,256-397,932), 11 had HIV RNA <20 c/mL after a mean 27.4 months of follow-up (range 12–33). Mean PK level was 2,920 ng/mL (1,268–4,562). One patient had virological failure after 14 months (HIV RNA 39,300 copies/mL); no mutations were detected and after introduction of DRV/r 600 mg b.i.d., he returned aviremic. All switched patients maintained HIV RNA suppression (<20 c/mL) for a mean of 32.8 months (range 21-54). PK level was available for one patient only (Ctrough 3,442 ng/mL). Of the treatment-experienced patients (mean baseline HIV RNA 24,167 log10 copies/mL, range 112–111,426), five maintained HIV RNA suppression for a mean of 46.2 months (range 31–67). One patient interrupted HAART for three months and then restarted it, the latest HIV RNA level being 628 copies/mL after five weeks of therapy. One patient failed after 42 months (HIV RNA 3,930 copies/mL); after intensification (DRV/r 600 twice daily), he returned aviremic. PK levels were available for three patients (mean 2,502 ng/mL; range 844–4,518).

**Conclusions:**

In this pilot study of 26 patients, use of DRV/r at 600/100 mg OD dose led to sustained HIV RNA suppression in 23 patients with acceptable PK exposures to DRV. Large non-inferiority trials are warranted to establish its efficacy.
